# Liquid Chromatography-High-Resolution Mass Spectrometry-Based In Vitro Toxicometabolomics of the Synthetic Cathinones 4-MPD and 4-MEAP in Pooled Human Liver Microsomes

**DOI:** 10.3390/metabo11010003

**Published:** 2020-12-23

**Authors:** Sascha K. Manier, Florian Schwermer, Lea Wagmann, Niels Eckstein, Markus R. Meyer

**Affiliations:** 1 Department of Experimental and Clinical Toxicology, Institute of Experimental and Clinical Pharmacology and Toxicology, Center for Molecular Signaling (PZMS), Saarland University, 66421 Homburg, Germany; Sascha.Manier@uks.eu (S.K.M.); florian.schwermer@hotmail.de (F.S.); Lea.Wagmann@uks.eu (L.W.); 2 Applied Pharmacy, Campus Pirmasens, University of Applied Sciences Kaiserslautern, 66953 Pirmasens, Germany; niels.eckstein@hs-kl.de

**Keywords:** untargeted metabolomics, 4-MPD, 4-MEAP, HPLC-HRMS/MS, metabolism

## Abstract

Synthetic cathinones belong to the most often seized new psychoactive substances on an international level. This study investigated the toxicometabolomics, particularly the in vitro metabolism of 2-(methylamino)-1-(4-methylphenyl)-1-pentanone (4-MPD) and 2-(ethylamino)-1-(4-methylphenyl)-1-pentanone (4-MEAP) in pooled human liver microsomes (pHLM) using untargeted metabolomics techniques. Incubations were performed with the substrates in concentrations ranging from 0, 12.5, and 25 µM. Analysis was done by means of high-performance liquid chromatography coupled to high-resolution mass spectrometry (HPLC-HRMS/MS) in full scan only and the obtained data was evaluated using XCMS Online and MetaboAnalyst. Significant features were putatively identified using a separate parallel reaction monitoring method. Statistical analysis was performed using Kruskal-Wallis test for prefiltering significant features and subsequent hierarchical clustering, as well as principal component analysis (PCA). Hierarchical clustering or PCA showed a distinct clustering of all concentrations with most of the features *z*-scores rising with the concentration of the investigated substances. Identification of significant features left many of them unidentified but revealed metabolites of both 4-MPD and 4-MEAP. Both substances formed carboxylic acids, were hydroxylated at the alkyl chain, and formed metabolites after combined hydroxylation and reduction of the cathinone oxo group. 4-MPD additionally formed a dihydroxy metabolite and a hydroxylamine. 4-MEAP was additionally found reduced at the cathinone oxo group, *N*-dealkylated, and formed an oxo metabolite. These findings are the first to describe the metabolic pathways of 4-MPD and to extend our knowledge about the metabolism of 4-MEAP. Findings, particularly the MS data of the metabolites, are essential for setting up metabolite-based toxicological (urine) screening procedures.

## 1. Introduction

Synthetic cathinones, derived from naturally occurring alkaloids of *Catha edulis* (Vahl) Forssk. ex Endl., are one of the biggest groups of new psychoactive substances (NPS). Mainly during the last two decades, a continuous flow of novel synthetic cathinones was seized by customs all over the world [1]. As classified by Simmler et al. [2], cathinones have a distinct but diverse pharmacology, which allow the deduction of structure–activity relationships on the one hand but leave an uncertainty about the pharmacology of new cathinones on the other hand.

**Figure 1:**
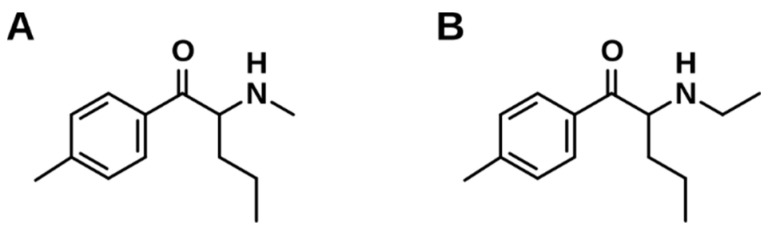
Chemical structures of the investigated synthetic cathinones. (**A**) = 4-MPD and (**B**) = 4-MEAP.

Besides their often-unknown pharmacological potency, a widely occurring problem is the false declaration of NPS sold online. 2-(Methylamino)-1-(4-methylphenyl)-1-pentanone (4-MPD, Figure 1A) is a prime example as it occurred the first time on the markets during the last decade. Previously published studies performed by Hamby et al. [3] [4] revealed a false declaration of 4-MPD, which turned out to be 2-(ethylamino)-1-(4-methylphenyl)-1-pentanone (4-MEAP, Figure 1B) instead.

4-MEAP was first reported to the Early Warning System of the European Monitoring Centre for Drugs and Drug Addiction (EMCDDA) by Luxembourg in January 2014 [4]. Distinct pharmacological data has not yet been reported for 4-MPD and 4-MEAP, but two case reports of misuse were published for 4-MEAP. Both individuals showed symptoms of increased dopaminergic and adrenergic activity including severe agitation and tachycardia but no signs of serotonergic activity such as mydriasis, hypertension, or pyrexia [5]. This is in accordance with known structure-activity relationships [2]. In 2019, another fatal case describing co-misuse of 4-MEAP was reported [6].

In vitro metabolism studies using pooled human liver microsomes (pHLM) coupled with in silico biotransformation predictions were additionally performed. pHLM is one of the most commonly applied metabolism models for the investigation of the metabolism of drugs [7]. Several earlier publications showed that this model is also well suited for the investigation of the metabolism of cathinone-based drugs of abuse [8,9]. These incubations revealed three metabolites in vitro (*N*-deethyl-, dihydro-, and dihydro-HO-4-MEAP) of which two metabolites (*N*-deethyl- and dihydro-4-MEAP) were confirmed in post-mortem human urine. Concerning 4-MPD, no case reports were published so far.

Untargeted metabolomics (UM) represents an approach to perform analyses of the metabolome by using computational peak detection and subsequent statistical evaluation across multiple study groups [10,11,12]. Previous studies revealed that UM is well suited to investigate the toxicometabolomics of NPS in vitro [12,13,14,15]. Elucidating the metabolism of emerging compounds such as NPS is a prerequisite for setting toxicological screening procedures e.g., in case of suspected intoxications or abuse [16,17]. The present study aimed, therefore, to first synthesize 4-MPD as well as 4-MEAP and afterwards elucidate their toxicometabolomics, particularly their metabolism in pooled human liver microsomes by means of liquid chromatography coupled to high-resolution Orbitrap mass spectrometry and UM.

## 2. Results and Discussion

### 2.1. Infrared Spectroscopy

Raw spectra in text format and the R script used to plot them can be found on GitHub (github.com/saskema/4mpd4meapphlm). Infrared spectroscopy (IR) is a valuable technique for the determination of functional groups and differentiation between similar substances by comparing fingerprinting regions. In direct comparison, both infrared spectra showed high similarity in peak location as expected (Figures S1 and S2). Strong aryl-carbonyl stretches (4-MPD: 1686 cm^−1^, 4-MEAP: 1689 cm^−1^) and an overlapping region of aliphatic and aromatic C-H stretching and amine salt NH_2_^+^ stretching (3000–2600 cm^−1^) represent the most characteristic bands for cathinone salts. The distinct single NH_2_^+^ deformation band at 1606 cm^−1^ additionally confirms the presence of secondary amine salts [18]. No IR data was published for 4-MEAP before but wavenumbers for 4-MPD were in accordance with previous publications [19,20]. Functional groups are identical and therefore the high similarity found in the spectra was not surprising. Differentiation of both substances was only achieved by subtle differences in intensity and peak position in the fingerprinting region.

Concerning the identity and purity of the compounds, the IR analysis assured that both compounds contain none to neglectable amounts of impurities, since 4-MPD matched exactly the above-mentioned reference spectra. 4-MEAP merely differs in its alkyl substitution at the amine moiety leading to minimal expectable variation concerning the IR-spectrum, such as the ratio of the peaks at 1467 and 1452 cm^−1^, indicating aliphatic C-H bending, as well as the intensity of the peaks at 2785 and 2728 cm^−1^ indicating C-H stretching. Additionally, due to the in-house synthesis of both compounds, the occurrence of highly similar structural isomers is highly unlikely.

### 2.2. Untargeted Metabolomics

**Figure 2:**
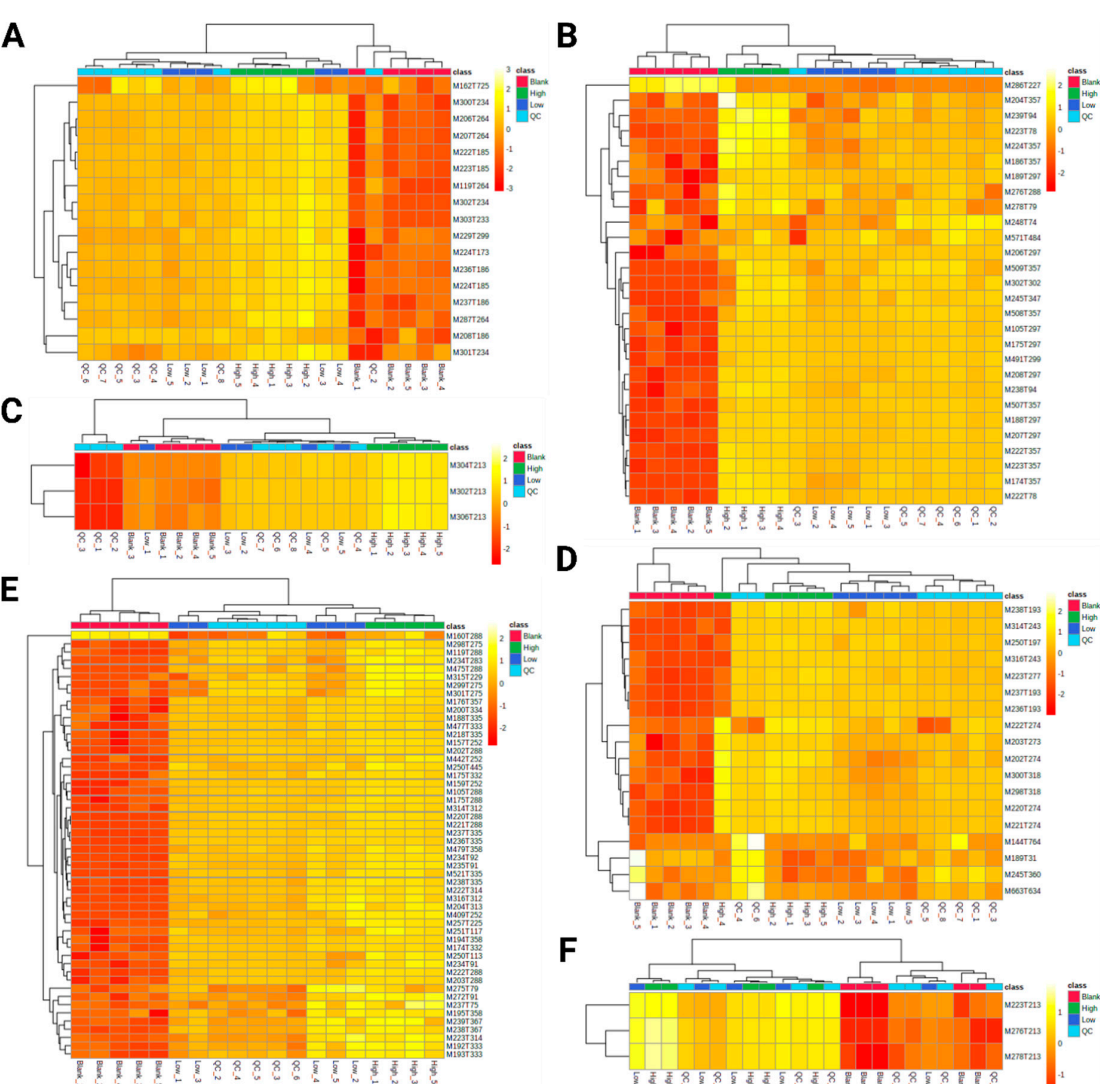
Heatmaps containing *z*-scores of the features’ abundances after hierarchical clustering. (**A**) 4-MPD, reversed phase chromatography, positive ionization mode; (**B**) 4-MPD, normal phase chromatography, positive ionization mode; (**C**) 4-MPD, normal phase chromatography, negative ionization mode. (**D**) 4-MEAP, reversed phase chromatography, positive ionization mode; (**E**) 4-MEAP, normal phase chromatography, positive ionization mode; (**F**) 4-MEAP, normal phase chromatography, negative ionization mode.

**Figure 3:**
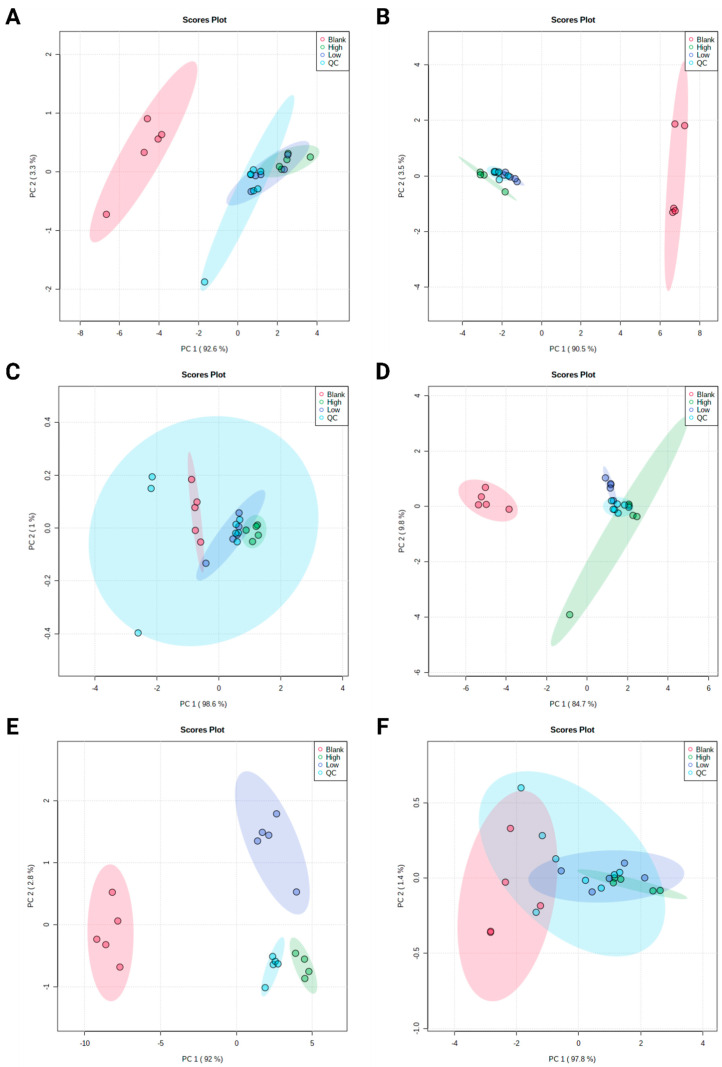
Scores of principal component analysis. (**A**) 4-MPD, reversed phase chromatography, positive ionization mode; (**B**) 4-MPD, normal phase chromatography, positive ionization mode; (**C**) 4-MPD, normal phase chromatography, negative ionization mode. (**D**) 4-MEAP, reversed phase chromatography, positive ionization mode; (**E**) 4-MEAP, normal phase chromatography, positive ionization mode; (**F**) 4-MEAP, normal phase chromatography, negative ionization mode, QC = pooled quality control sample injections.

Files and metadata reported in this paper are available via Metabolights and the study identifier MTBLS2218. R commands as obtained from MetaboAnalyst to reproduce statistical evaluation can be found on GitHub (github.com/saskema/4mpd4meapphlm). Heatmaps containing *z*-scores of the features’ abundances and dendrograms after hierarchical clustering are displayed in Figure 2, scores after PCA in Figure 3, and loadings after PCA are shown in Figure S3.

The following samples were determined as outlier and needed to be removed from further evaluations of the study. QC 1 (quality control injection 1, a pooled sample obtained by mixing an aliquot of all samples) after analysis using reversed phase chromatography and positive ionization mode, High 5 (sample 5 from group High) and QC 8 after analysis using normal phase chromatography and positive ionization mode (incubations of 4-MPD). QC 2 after analysis using reversed phase chromatography and positive ionization mode, High 4 and QC 1, 7, and 8 after analysis using normal phase chromatography and positive ionization mode (incubations of 4-MEAP). The decision for each sample was made by inspecting both the hierarchical clustering, as well as their clustering in PCA scores. Further inspection of the excluded samples revealed increased instrument variability. However, the presented data is shown to be consistent and still revealed significant features as discussed in the following sections.

Concerning 4-MPD, the hierarchical clustering mostly revealed a high distance of samples from group Blank to those from other groups in analyses using positive ionization mode (Figure 2A,B). While samples from group High did also form an isolated cluster, samples from group Low and QC overlapped with one sample. This overlap can be explained by the fact that the sample that forms group QC after multiple injections is pooled from each of the other samples. Given the equally increased concentration of 4-MPD from group Blank to group High, the concentration of analytes in group QC approximately corresponds to that of group Low. These results mainly correspond by the *z*-scores of the features’ abundances given in the heatmap. Merely M162T725 in Figure 2A showed *z*-scores that do not seem to correlate with sample groups. Given the fact, that its *z*-scores are not equally distributed throughout group QC, it is most likely this that these observations are caused by batch effects [21]. Regarding the analysis using normal phase chromatography and negative ionization mode, samples of group Blank and High are clustered as described above (Figure 2C). However, since only three significant features were found and the investigated synthetic cathinones with their high concentrations are not sufficiently ionized in negative mode, their clustering is less clear compared to analysis in positive mode.

The results of 4-MEAP after hierarchical clustering (Figure 2D–F) are more or less the same as described for 4-MPD. It is notable that the features M144T764, M189T31, M245T360, and M663T634 after analysis using reversed phase chromatography and positive ionization mode (Figure 2D) have comparatively high *z*-scores in group QC. This might be explained by ion enhancement, due to the pooling of all samples to obtain samples of group QC [22].

The results of PCA corresponded to those obtained after hierarchical clustering, with groups Blank and High forming distinct clusters and group Low and group QC overlapping (Figure 3A–F). Nevertheless, the overlap of one QC sample with group Blank in Figure 2A was not observed after PCA (Figure 3A).

### 2.3. Identification of Significant Features

Results of the identification of significant features are summarized in Tables S2–S5. MS^2^ spectra of significant features, available as indicated in Tables S2–S5 in the supplementary data, can as well be found on GitHub (github.com/saskema/4mpd4meapphlm) in mzXML format. However, for quick access and interpretation, all spectra of 4-MPD, 4-MEAP, and their metabolites can be found in Figures S4 and S5 with their tentative structures and proposed fragmentation patterns. Isotopes, adducts, and artifacts that were annotated by CAMERA were not further analyzed. Each of the remaining features were analyzed using the parallel reaction monitoring (PRM) method as described above. Regarding their identification level as proposed by Sumner et al. [23], all identified metabolites of 4-MPD and 4-MEAP were putatively identified by deduction from the spectra of the parent compounds, leading to a classification level of 3. All of the following masses used to describe ions and fragments are theoretical masses.

#### 2.3.1. 4-MPD

In total, 5 metabolites, 8 artifacts, and 11 isotopes could be putatively identified (Tables S2 and S3). The fragmentation pattern of 4-MPD with the protonated molecular ion at *m/z* 206.1539 (C_13_H_20_ON) was in accordance with a previous characterization by Apirakkan et al. [24] (M206T264/M206T297 in Figure S4). Water loss (−18.0100 u) after elimination of the cathinone oxo group resulted in the fragment ion at *m/z* 188.1433 (C_13_H_18_N), further sigma bond cleavage led to the fragment ion at *m/z* 146.0964 (C_10_H_12_N). Elimination of the amine moiety of 4-MPD resulted in the fragment ion at *m/z* 175.1117 (C_12_H_15_O). Additionally, the fragment after benzyl cleavage with fragment ion at *m/*z 105.0707 (C_8_H_9_) and the rather unspecific tropylium ion with *m/z* 91.0542 (C_7_H_7_) were characteristic for this substance.

The metabolite formed after hydroxylation of the alkyl chain of 4-MPD (M222T357 in Figure S4) with protonated *m/z* 222.1488 (C_13_H_20_O_2_N) was putatively identified by a second water loss resulting in the fragment ion with *m/z* 186.1277 (C_13_H_16_N). The tropylium ion with *m/z* 91.0542 (C_7_H_7_) ruled out a hydroxylation at the phenyl ring, making an alkyl hydroxylation most likely. *N*-oxidation of 4-MPD (M222T78 in Figure S4) resulted in the same precursor ion with *m/z* 222.1488 (C_13_H_20_O_2_N). The conclusion of an *N*-oxidation rather than a hydroxylation of 4-MPD was drawn due to the fact that a second water loss was not observed and the fragment ion at *m/z* 162.0917 (C_10_H_12_ON) ruled out a hydroxylation of the alkyl chain. Additionally, the fragment ion with *m/z* 119.0491 (C_8_H_7_O) formed after benzylic scission ruled out a hydroxylation of the phenyl ring. At last, the metabolite formed after *N*-oxidation eluted earlier than 4-MPD after using normal phase chromatography, which is in accordance with similar observations concerning hydroxyl amines using reversed phase chromatography [25]. All other metabolites were following the same fragmentation pattern and thus putatively identified accordingly.

#### 2.3.2. 4-MEAP

In total, 6 metabolites, 10 artifacts, 12 isotopes, and 1 adduct could be putatively identified (Tables S4 and S5). The fragmentation pattern of 4-MEAP with the protonated molecular ion at *m/z* 220.1695 (C_14_H_22_ON) (M220T274/M220T288 in Figure S5) was in accordance with Hamby et al. [3]. Similar to 4-MPD an initial water loss after the elimination of the cathinone oxo group led to the formation of the fragment ion with *m/z* 202.1590 (C_14_H_20_N). Further sigma bond cleavage led to the fragment ion with *m/z* 160.1120 (C_11_H_14_N) and a benzylic scission led to formation of the fragment ion with *m/z* 105.0706 (C_8_H_9_). Again, the formation of a tropylium ion with *m/z* 91.0542 (C_7_H_7_) was observed. Most of the metabolites of 4-MEAP were identified accordingly to 4-MPD and their fragmentation pattern was the same as observed for 4-MPD. However, some metabolic pathways of 4-MEAP were not observed for 4-MPD. The metabolite formed after *N*-deethylation (M192T258/M192T333 in Figure S4) with protonated *m/z* 192.1382 (C_12_H_18_ON) was identified by the fragment ion with *m/z* 132.0807 (C_9_H_8_N_2_). This fragment represents the parent ion after water elimination and further alkyl elimination. Compared to the spectrum of 4-MEAP, the fragment is shifted by C_2_H_4_ (−28.0313 u), while the fragment formed after water elimination and benzylic scission with *m/z* 105.0698 (C_8_H_9_) and the tropylium ion with *m/z* 91.0542 (C_7_H_7_) remained the same. Reduction of the cathinone oxo group (M222T277/M222T314 in Figure S4) resulted in the metabolite with the protonated *m/z* 222.1852 (C_14_H_24_O_2_N). The reduction was putatively identified by the fragment ion with *m/z* 204.1746 (C_14_H_22_N) formed after elimination of water and the fragment ion with *m/z* 161.1199 (C_11_H_15_N) formed after elimination of the alkyl chain. The fact that both fragments are shifted by at least one hydrogen implies that the initial elimination of water did not result in the formation of an azirine ring as proposed by Franski et al. [26] earlier. At last, the formation of an oxo group (M234T92 in Figure S4) was observed leading to the metabolite with protonated *m/z* 234.1488 (C_14_H_20_O_2_N). In contrast to the spectra to all of the other metabolites, the oxo metabolite did not perform an initial elimination of the cathinone oxo group. Instead, the fragment ion after benzylic scission with *m/z* 119.0491 (C_8_H_7_O) is present with the highest abundance except for the parent ion. This might be explained by assuming the oxo group to be in γ-position to the cathinone oxo group. It would lead to a hydrogen bond with the protonated amine moiety that further prevents the elimination of the cathinone oxo group.

### 2.4. Proposed Metabolic Pathways

**Figure 4:**
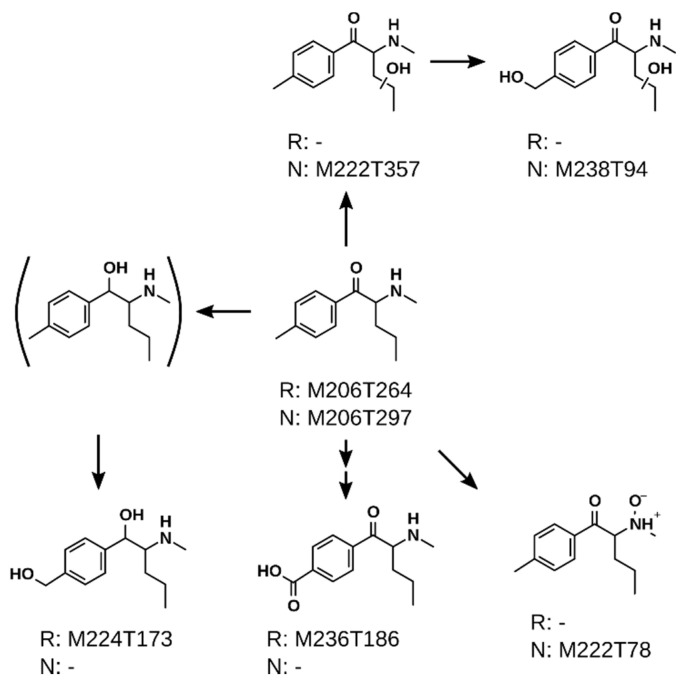
Proposed metabolic pathways of 4-MPD in pHLM. Every metabolite is labeled by its feature identifier as detected within untargeted metabolomics analysis. R = reversed phase chromatography; N = normal phase chromatography. Structure in brackets is a postulated intermediate metabolite.

**Figure 5:**
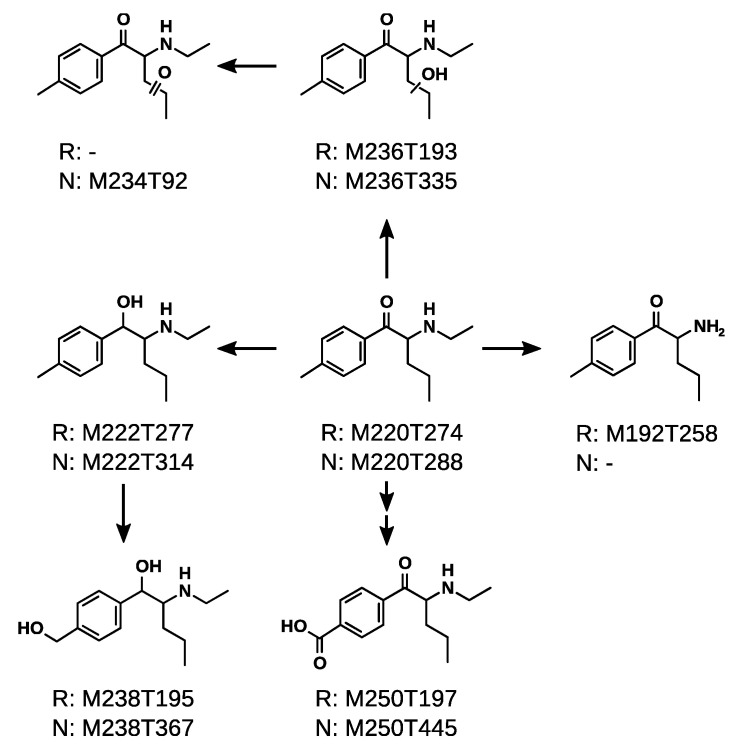
Proposed metabolic pathways of 4-MEAP in pHLM. Every metabolite is labeled by its feature identifier as detected within untargeted metabolomics analysis. R = reversed phase chromatography; N = normal phase chromatography.

The metabolic pathways of 4-MPD and 4-MEAP are shown in Figure 4 and Figure 5. Both compounds share several metabolic steps such as hydroxylation of the alkyl chain (M222T357 in Figure 4 and M236T193/M236T335 in Figure 5), formation of a carboxylic acid (M236T186 in Figure 4 and M250T197/M250T445 in Figure 5) and reduction of the cathinone oxo group in combination with hydroxylation of the benzylic position (M224T173 in Figure 4 and M238T195/M238T367 in Figure 5). In case of 4-MPD, additional *N*-oxidation (M222T78 in Figure 4) as well as hydroxylation of the alkyl chain in combination with hydroxylation of the benzylic position (M238T94 in Figure 4) was detected. Although being a commonly detected metabolite for cathinones, the intermediate reduction of the cathinone oxo group was not detected by the peak picking algorithm [6,27]. Manual inspection of the extracted ion chromatograms revealed that it is presumably overlapped with a ^13^C_2_-isotope at 208.1657 (C_12_^13^C_2_H_21_NO), which was most likely not properly resolved from that of the dihydro metabolite with its protonated ion at *m/z* 208.1701 (C_13_H_22_NO) (see Figure S6). This observation was already described earlier with other synthetic cathinones [12,28]. Concerning 4-MEAP, an additional formation of an oxo group at the alkyl chain was detected (M234T92 in Figure 5), as well as dealkylation of the amine moiety (M192T258 in Figure 5), and reduction of the cathinone oxo group (M222T277/M222T314 in Figure 5).

These metabolic steps are in accordance with earlier publications investigating the metabolism of similar compounds such as 4-chloroethcathinone, *N*-ethylnorpentylone, *N*-ethylhexedrone (NEH), and 4-fluoro-alpha-pyrrolidinohexiophenon in pooled human liver S9 fraction, human blood, or human urine [27]. *N*-Dealkylation, reduction of the cathinone oxo group, and hydroxylation of the alkyl chain were detected for NEH amongst others in this study. The formation of carboxylic acid in benzylic position was also described by Olesti et al. earlier [29]. Concerning 4-MEAP, its metabolism was already described by Benedicte et al. [6]. They detected *N*-deethylation, as well as benzylic hydroxylation and further reduction of the cathinone oxo group. However, hydroxylation of the alkyl chain, further oxidation forming an oxo group, as well as formation of a carboxylic acid in benzylic position are metabolic pathways were not found.

It is notable that 4-MPD did not form a dealkyl metabolite although this step is described as one of the most common metabolic pathways for synthetic cathinones [28]. This finding excludes the occurrence of a common metabolite of 4-MPD and 4-MEAP which might be misleading in some toxicological analyses. However, one has to remember that the here presented data are in vitro data, making the occurrence of a dealkyl metabolite of 4-MPD in vivo still possible. Additionally, unlike other NPS such as synthetic cannabinoids, that are extensively metabolized and often form structurally identical metabolites, synthetic cathinones are also excreted unchanged. The parent mass of the feature M192T304 (Table S3) might indicate to a possible dealkyl metabolite of 4-MPD, although the abundance of obtained MS/MS spectra was too low to allow a final conclusion.

## 3. Materials and Methods

### 3.1. Chemicals and Reagents

All chemicals used for syntheses were obtained from Sigma Aldrich (Taufkirchen, Germany). Hydrochloric acid gas in 2-propanol was prepared by gassing isopropanol with hydrogen chloride (generated from the reaction between conc. H_2_SO_4_ and sodium chloride) in the laboratory. NADP-Na_2_, acetonitrile (LC-MS grade), and methanol (LC-MS grade) were obtained from VWR (Darmstadt, Germany), MgCl_2_, K_2_HPO_4_, KH_2_PO_4_, superoxide dismutase, isocitrate dehydrogenase, isocitrate, ammonium formate, ammonium acetate, and formic acid from Sigma (Taufkirchen, Germany). Water was purified with a Millipore filtration unit (18.2 Ω × cm water resistance). pHLM (pool of 25 donors, 20 mg microsomal protein/mL) were obtained from Corning (Amsterdam, The Netherlands). After delivery, the pHLM were aliquoted, snap-frozen in liquid nitrogen, and stored at −80 °C until use.

### 3.2. Synthesis of 2-(Methylamino)-1-(4-Methylphenyl)-1-Pentanone (4-MPD) and 2-(Ethylamino)-1-(4-Methylphenyl)-1-Pentanone (4-MEAP)

A universally applicable synthesis pathway to obtain analogues of cathinone was used to synthesize 4-MPD and 4-MEAP in three steps. The first step was a Friedel-Crafts acylation under standard conditions using toluene and valeroyl chloride which formed the phenone intermediate [30]. The second step was an alpha-carbonyl bromination which formed the brominated phenone from the previously obtained phenone intermediate and *N*-bromosuccinimide, followed by substitution with the appropriate alkylamine in the third step [31]. Alkylamine addition was performed whilst cooling to prevent condensation and possible pyrazine formation side reactions. Thereafter, hydrochloride salts were obtained using a solution of hydrochloric acid gas dissolved in 2-propanol. All solvents used herein were dried with 10% *m/v* 3Å molecular sieves for at least 24 h prior to use.

To obtain 1-(4-methylphenyl)pentane-1-one, toluene (50 mmol) in dichloromethane (5 mL) was added drop wise to a mixture of aluminum chloride (55 mmol), valeroyl chloride (52 mmol), and dichlormethane (20 mL) under stirring and cooling in an ice-bath. After all the toluene solution was added, the resulting reaction mixture was stirred for further 2 h. The reaction mixture was poured on crushed ice, the organic phase was separated, and the aqueous phase was re-extracted using dichloromethane (1 × 20 mL) and the combined organic phases were sequentially washed with water, 1 M sodium hydroxide solution and brine, and dried over anhydrous sodium sulfate. The solvent was evaporated to yield the product as light amber oil (7.39 g, 42 mmol, 84% based on toluene). The isolated product was used without further purification in the next step.

In order to obtain 2-bromo-1-(4-methylphenyl)pentane-1-one, 1-(4-methylphenyl)pentan-1-one (42 mmol) was dissolved in acetonitrile (10 mL). *N*-Bromosuccinimide (47 mmol) and *para*-toluenesulfonic acid monohydrate (4.2 mmol) were added and the reaction mixture was warmed to 60 °C water bath temperature and allowed to stir for 2 h. After cooling to room temperature, water (20 mL), dichloromethane (30 mL), and 1 M sodium hydroxide solution (20 mL) were added, the solution was stirred for 10 min and the organic phase was separated and sequentially washed with 1M sodium hydroxide solution and brine. The organic phase was dried over sodium sulfate, filtered on a Büchner funnel, and further used as is in solution.

The final product 2-(methylamino)-1-(4-methylphenyl)-1-pentanone (4-MPD) hydrochloride was obtained after dissolving methylamine hydrochloride (60 mmol) and NaOH (60 mmol) in water (6 mL) and slowly adding it to one half of the 2-bromo-1-(4-methylphenyl)pentan-1-one solution (21 mmol) with strong stirring at −10 °C. After all the methylamine solution had been added, the resulting reaction mixture was stirred for 1 h after which water (20 mL) was added and stirring was continued for another 10 min. The organic phase was separated and washed with water and brine, cooled in an ice bath, and titrated with hydrochloric acid gas dissolved in 2-propanol until neutral. After freezing overnight, the resulting product was filtered off, washed with cold anhydrous acetone, and dried to a final yield of 0.41 g (2.0 mmol, 10%) of the hydrochloride salt as a white solid.

4-MEAP was synthesized as described for 4-MPD from 2-bromo-1-(4-methylphenyl)pentan-1-one using ethylamine hydrochloride to obtain 0.35 g (1.6 mmol, 7.6%) of the hydrochloride salt as a white solid.

### 3.3. Infrared Spectroscopy Apparatus and Analysis

IR spectra of the hydrochloride salts were acquired by solid KBr disks (ratio 1:300 *w/w*, substance/KBr, applied pressure 9×10^4^ N) using a Bruker (Rheinstetten, Germany) IFS 66 v/S under vacuum (7 mbar). IR spectra were recorded in a wavenumber range of 4000–600 cm^−1^ with a wavelength resolution of 4 cm^−1^ and 32 scans per spectrum. IR data was handled using Bruker OPUS 5.5.

### 3.4. HPLC-HRMS/MS Apparatus

The analysis of UM samples was performed as described by Manier et al. [12] using a high-performance liquid chromatography system coupled to a high-resolution mass spectrometer (HPLC-HRMS/MS) consisting of a Thermo Fisher Scientific (TF, Dreieich, Germany) Dionex UltiMate 3000 RS pump coupled to a TF Q-Exactive Plus mass spectrometer. The UltiMate 3000 RS system consisted of a degasser, a quaternary pump, an autosampler, and an analytical column heater (column temperature 40 °C). Mass calibration was done prior to analysis according to the manufacturer’s recommendations using external mass calibration. Additionally, before each experiment, the spray shield and capillary were cleaned. The performance of the column and the mass spectrometer was tested using a mixture as described by Maurer et al. [32] prior to every experiment. Gradient reversed phase elution was performed on a TF Accucore Phenyl-Hexyl column (100 × 2.1 mm, 2.6 µm). The mobile phases consisted of 2 mM aqueous ammonium formate containing formic acid (0.1%, *v/v*) and acetonitrile (1%, *v/v*, pH 3, eluent A), as well as 2 mM ammonium formate in acetonitrile/methanol (1:1, *v/v*) containing formic acid (0.1%, *v/v*) and water (1%, *v/v*, eluent B). The flow rate was set from 1–10 min to 500 µL/min and from 10–13.5 min to 800 µL/min using the following gradient: 0–1.0 min 99% A, 1–10 min to 1% A, 10–11.5 min hold 1% A, 11.5–13.5 min hold 99% A. For normal phase elution, a Macherey-Nagel (Düren, Germany) HILIC Nucleodur column (125 × 3 mm, 3 µm) was used. The mobile phases consisted of 200 mM aqueous ammonium acetate (eluent C) and acetonitrile containing formic acid (0.1%, *v/v*, eluent D). The flow rate was set to 500 µL/min using the following gradient: 0–1 min 2% C, 1–5 min 20% C, 5–8.5 min 60% C, 8.5–10 min hold 60% C, 10–12 min hold 2% C. The injection volume for every analysis was 1 µL. The Q-Exactive Plus was equipped with a heated electrospray ionization source (HESI-II) and was operated in both, positive and negative ionization mode. The spray voltage was 3.50 kV, capillary temperature, 320 °C; heater temperature, 320 °C; S-lens RF level, 50.0; sheath gas flow rate, 60 AU; auxiliary gas flow rate, 10 AU and sweep gas, 3 AU. Mass spectrometry for UM was performed according to a previously optimized workflow by Manier et al. [33] using full scan (FS) only. The resolution was 140,000 full width at half maximum (FWHM) at mass to charge ration (*m/z*) 200; microscans, 1; automatic gain control target, 5 × 10^5^; maximum injection time, 200 ms; scan range, *m/z* 50–750; polarity negative or positive and spectrum data type centroid. Thermo Fisher Scientific (TF, Dreieich, Germany) Xcalibur version 4.0.27.19 software was used for data acquisition and manipulation. The analysis was performed using a randomized sequence order with five injections of pure methanol (Phenyl-Hexyl column) or eluent D (HILIC column) samples at the beginning of the sequence for apparatus equilibration, followed by five injections of the pooled QC sample. Additionally, one QC injection was performed every five samples.

Statistically significant features were putatively identified using parallel reaction monitoring (PRM): resolution, 70,000 FWHM; microscans, 1; automatic gain control target, 5 × 10^5^; maximum injection time, 200 ms; isolation window, 0.4 *m/z*; normalized collision energy, 10, 20, and 40 eV; spectrum data type, centroid.

### 3.5. Microsomal Incubations Using pHLM

Microsomal incubations were performed as initially described by Welter et al. [34] with some modifications. Stock solutions of the investigated substances were prepared at concentrations of 125 and 62.5 µM in 100 mM phosphate buffer. The incubation mixture of each sample had a total volume of 50 µL and contained 90 mM phosphate buffer, 200 U/mL superoxide dismutase, 5 mM isocitrate, 5 mM MgCl_2_, 1.2 mM NADP^+^, 0.5 U/mL isocitrate dehydrogenase, 1 mg protein/mL pHLM, and 25 µM (further referred to as group High), 12.5 µM (further referred to as group Low), or 0 µM (further referred to as group Blank) substrate. Each concentration was prepared in five replicates. The substrate was added after preincubation of the incubation mixture in the orbital shaker (10 min, 37 °C, 200 rpm) to incubate for another 60 min (37 °C, 200 rpm). Incubations were stopped by addition of 50 µL ice cold acetonitrile and centrifugation for 2 min at 14,000 U/min. The supernatant was transferred to an MS vial and a pooled QC sample was prepared using 10 µL of each prepared sample. All samples were analyzed using HPLC-HRMS/MS as described above.

### 3.6. Data Processing for Untargeted Metabolomics

The proprietary TF raw data format files were converted to the open data format mzXML using ProteoWizard’s MSConvert (version 3.0.1) [35]. Subsequently, QC files were used for peak detection parameter optimization as described by Manier et al. [33]. Peak picking parameters that were used in this study are summarized in Table S1. Preprocessing and statistical evaluation was conducted using XCMS Online (version 3.7.1) and MetaboAnalyst (via https://www.metaboanalyst.ca/) (version 4.0). The converted MS data sets of Blank, Low, High, and QC were uploaded, and the parameters were adjusted according to the optimized values generated through the algorithm. Afterwards, features with valid extracted ion chromatograms and *p*-values lower than or equal to 0.01 after Kruskal-Wallis analysis were kept. Chromatograms were assumed valid when the peak detection integrated gaussian-like chromatographic peaks rather than arbitrary baseline fluctuation. Samples were also checked for within group outliers and removed from the study in case of high deviation. The obtained feature table was subsequently uploaded to MetaboAnalyst for further statistical evaluation. In MetaboAnalyst, missing value estimation was skipped because in no case missing values were present. Data filtering was skipped as well because in every case, less than 5000 features were submitted and therefore a need to compress the data was not given. After log transformation, the data set was submitted to multivariate statistical analysis. Hierarchical clustering was performed using Euclidian distances and complete clustering. Color contrast in the corresponding heatmap was set to “Heat Color”, samples were not reorganized, and normalized data was selected to be displayed. Additionally, features were autoscaled for this analysis. Finally, a PCA was performed to inspect the samples clustering in the corresponding score plots and the features’ influence on the clustering in the corresponding loading plots. The data set was not scaled prior to PCA. Names of the features were adopted from XCMS Online using “M” followed by the rounded mass and “T” followed by the retention time in seconds (e.g., “M206T264” as given in Table S2 for protonated 4-MPD at *m/z* 206.1543 and a retention time of 264 s using reversed phase chromatography).

### 3.7. Identification of Significant Features

MS^2^ spectra were recorded using the above-mentioned PRM method to allow identification of significant features. Individual spectra were exported after subtracting the baseline left and right of the peak. After conversion to mzXML format using ProteoWizard’s MS Convert, spectra were imported to NIST MSSEARCH version 2.3. A library search for identification was conducted using the following settings: spectrum search type, identity (MS/MS); precursor ion *m/z*, in spectrum; spectrum search options, none; presearch, off; other options, none. MS/MS search was conducted using the following settings: precursor tolerance, ±5 ppm; product ion tolerance, ± 10 ppm; ignoring peaks around precursor, ± *m/z* 1. The search was conducted by using the following libraries: NIST 14 (nist_msms and nist_msms2 sublibraries) and Wiley METLIN Mass Spectral Database. Metabolites of the investigated NPS were putatively identified by interpreting their spectra in comparison to those of the parent compounds.

## 4. Conclusions

Due to the high abundance and fluctuation of the NPS market, many NPS still lack substantial pharmacological and toxicological characterization. We described the successful synthesis the two cathinones 4-MPD and 4-MEAP and elucidated their in vitro metabolism in pHLM by using untargeted toxicometabolomics in combination LC-HRMS/MS. For both, 4-MPD and 4-MEAP, five and six metabolites could be putatively identified, respectively. The discussed analytical findings are essential to allow their detection in (urine) screening procedures e.g., in case of intoxications.

## Data Availability

Files and metadata reported in this paper are available via Metabolights and the study identifier MTBLS2218. IR-spectra in text format and the R script used to plot them can be found on GitHub (github.com/saskema/4mpd4meapphlm). R commands as obtained from MetaboAnalyst to reproduce statistical evaluation can also be found on GitHub, as well as MS^2^ spectra of significant features, available as indicated in Tables S2–S5, in mzXML format.

## Supplementary Materials

The following are available online at https://www.mdpi.com/2218-1989/11/1/3/s1: Table S1: Peak picking and alignment parameters used for preprocessing of data concerning 4-MPD and 4-MEAP, Table S2: Significant features of 4-MPD detected using reversed phase chromatography. Table S3: Significant features of 4-MPD detected using normal phase chromatography, Table S4: Significant features of 4-MEAP detected using reversed phase chromatography, Table S5: Significant features of 4-MEAP detected using normal phase chromatography, Figure S1: FT-IR spectrum of 4-MPD HCl (KBr pellet), Figure S2: FT-IR spectrum of 4-MEAP HCl (KBr pellet), Figure S3: Loadings of principal component analysis, Figure S4: LC–HRMS/MS spectra of 4-MPD and its metabolites sorted by mass of their protonated molecule and retention time, Figure S5: LC–HRMS/MS spectra of 4-MEAP and its metabolites sorted by mass of their protonated molecule and retention time, Figure S6: Extracted ion chromatogram of two peaks assumed to be 4-MPD isotope (13C2) and 4-MPD-M (dihydro-) taken from sample High 1 after analysis using reversed phase chromatography and positive ionization.
