# Supplementary material for: Liquid Chromatography-High-Resolution Mass Spectrometry-Based In Vitro Toxicometabolomics of the Synthetic Cathinones 4-MPD and 4-MEAP in Pooled Human Liver Microsomes
Source: Metabolites. 2020 Dec 23;11(1):3. doi: 10.3390/metabo11010003 (PMC7824391; doi:10.3390/metabo11010003)
Supplement: Supplementary file 1 [file metabolites-11-00003-s001.pdf]

Article

Liquid Chromatography-High-Resolution Mass Spectrometry-Based In Vitro Toxicometabolomics of the Synthetic Cathinones 4-MPD and 4-MEAP in Pooled Human Liver Microsomes

Sascha K. Manier [1], Florian Schwermer [1,2], Lea Wagmann [1], Niels Eckstein [2] and Markus R. Meyer [1,*]

[1] Department of Experimental and Clinical Toxicology, Institute of Experimental and Clinical Pharmacology and Toxicology, Saarland University, Center for Molecular Signaling (PZMS), 66421 Homburg, Germany; Sascha.Manier@uks.eu (S.K.M.); florian.schwermer@hotmail.de (F.S.); Lea.Wagmann@uks.eu (L.W.)

[2] Applied Pharmacy, University of Applied Sciences Kaiserslautern, Campus Pirmasens, 66953 Pirmasens, Germany; niels.eckstein@hs-kl.de

* Correspondence: markus.meyer@uks.eu; Tel.: +49-6841-12626438

Table S1. Peak picking and alignment parameters used for preprocessing of data concerning 4-MPD and 4-MEAP. R = reversed phase chromatography, N = normal phase chromatography, pos = positive, neg = negative, ppm = allowed ppm deviation of mass traces for peak picking, snthresh = signal to noise threshold, mzdiff = minimum difference in *m/z* for two peaks to be considered as separate, prefilter 1 = minimum of scan points, prefilter 2 = minimum abundance, bw = bandwidth for grouping of peaks across separate chromatograms. The numbers in brackets represent the chosen values in cases where manual adjustment was necessary except for bandwidth, where values had to be changed because XCMS Online only accepted integer values.

Substance	Column	Polarity	ppm	peak width (min)	peak width (max)	snthresh	mzdiff	prefilter 1	prefilter 2	bw
4-MPD	R	pos	2.5	6.8	21	86	0.008	1	100	1
4-MPD	R	neg	1.1	8.9	91	57	0.04	1	100	1.7 (2)
4-MPD	N	pos	1.9	8.9	93	100	0.01	1	100	1.3 (1)
4-MPD	N	neg	1.9	7.8	100	64 (100)	0.01	1	3500 (100000)	1.2 (1)
4-MEAP	R	pos	1.5	7.8	87	98	0.008	1	100	2
4-MEAP	R	neg	1.6	8.9	100	100	0.04	5	100	2
4-MEAP	N	pos	2.5	8.9	35	77	0.008	5	100	1.2 (1)
4-MEAP	N	neg	2.5	6.8	60	6 (100)	0.008	1 (5)	1700 (100000)	1.9 (2)

Table S2. Significant features of 4-MPD detected using reversed phase chromatography. Features ordered by ionization mode, mass per charge (*m/z*) and retention time. RT = retention time, CE = collision energy, pos = positive, neg = negative, x = available, / = not analyzed, n.d. = not detectable.

Ionization Mode	Feature	Identity	*m/z*	RT, s	Available MS^2 Spectrum, CE		
					10	20	40
pos	M119T264	4-MPD artifact $[M + H - C_6H_{15}N]^+$	119.0489	264	/	/	/
pos	M206T264	4-MPD	206.1543	264	x	x	x
pos	M207T264	4-MPD ^{13}C-isotope	207.1575	264	/	/	/
pos	M222T185	Unknown	222.1491	185	x	x	x
pos	M223T185	4-MPD-M (hydroxylamine) ^{13}C-isotope	223.1524	185	/	/	/
pos	M224T173	4-MPD-M (dihydro-HO-)	224.1647	173	x	x	x
pos	M224T185	4-MPD-M () $^{13}C_2$-isotope	224.1557	185	/	/	/
pos	M229T299	Unknown	229.1702	299	x	x	x
pos	M236T186	4-MPD-M (HOOC-)	236.1283	186	x	x	x
pos	M237T186	4-MPD-M (HOOC-) ^{13}C-isotope	237.1316	186	/	/	/
pos	M287T264	Unknown	287.2119	264	x	x	x
pos	M300T234	Unknown	300.0596	234	x	x	x
pos	M301T234	Unknown	301.0629	234	n.d.	n.d.	n.d.
pos	M302T234	Unknown	302.0575	234	n.d.	n.d.	n.d.
pos	M303T233	Unknown	303.0608	233	n.d.	n.d.	n.d.

Table S3. Significant features of 4-MPD detected using normal phase chromatography. Features ordered by mass per charge (*m/z*) and retention time. RT = retention time, CE = collision energy, pos = positive, neg = negative, x = available, / = not analyzed, n.d. = not detectable.

Ionization Mode	Feature	Identity	*m/z*	RT, s	Available MS^2 Spectrum, CE		
					10	20	40
pos	M105T297	4-MPD artifact $[M+H-C_5H_{11}ON]^+$	105.0703	297	/	/	/
pos	M174T357	4-MPD-M (HO-) artifact $[M+H-CH_4O_2]^+$	174.1277	357	/	/	/
pos	M175T297	4-MPD artifact $[M+H-CH_5N]^+$	175.1180	297	/	/	/
pos	M186T357	4-MPD-M (HO-) artifact $[M+H-H_4O_2]^+$	186.1277	357	/	/	/
pos	M188T297	4-MPD artifact $[M+H-H_2O]^+$	188.1434	297	/	/	/
pos	M189T297	4-MPD artifact $[M+H-H_2O]^+$ ^{13}C-isotope	189.1468	297	/	/	/
pos	M192T304	Unknown	192.1383	304	x	x	x
pos	M204T357	4-MPD-M (HO-) artifact $[M+H-H_2O]^+$	204.1384	357	/	/	/
pos	M206T297	4-MPD	206.1541	297	x	x	x
pos	M207T297	4-MPD ^{13}C-isotope	207.1574	297	/	/	/
pos	M208T297	4-MPD $^{13}C_2$-isotope	208.1606	297	/	/	/
pos	M222T357	4-MPD-M (HO-)	222.1490	357	x	x	x
pos	M222T78	4-MPD-M (hydroxylamine-)	222.1489	78	x	x	x
pos	M223T357	4-MPD-M (HO-) ^{13}C-isotope	223.1522	357	/	/	/
pos	M223T78	4-MPD-M (hydroxylamine-) ^{13}C-isotope	223.1522	78	/	/	/
pos	M224T357	4-MPD-M (HO-) $^{13}C_2$-isotope	224.1556	357	/	/	/
pos	M238T94	4-MPD-M (di-HO-)	238.1438	94	x	x	x
pos	M239T94	4-MPD-M (di-HO-) ^{13}C-isotope	239.1469	94	/	/	/
pos	M276T288	Unknown	276.1957	288	n.d.	n.d.	n.d.
pos	M278T79	Unknown	278.2117	79	n.d.	n.d.	n.d.
pos	M302T302	Unknown	302.0573	302	n.d.	n.d.	n.d.
neg	M302T213	Unknown	301.6827	213	n.d.	n.d.	n.d.
neg	M304T213	Unknown	303.6807	213	n.d.	n.d.	n.d.
neg	M306T213	Unknown	305.6788	213	n.d.	n.d.	n.d.
pos	M507T357	Unknown	507.2558	357	x	x	x
pos	M508T357	M507T357 ^{13}C-isotope	508.2594	357	/	/	/
pos	M509T357	M507T357 $^{13}C_2$-isotope	509.2514	357	/	/	/

Table S4. Significant features of 4-MEAP detected using reversed phase chromatography. Features ordered by ionization mode, mass per charge (*m/z*) and retention time. RT = retention time, CE = collision energy, pos = positive, neg = negative, x = available, / = not analyzed, n.d. = not detectable.

Ionization Mode	Feature	Identity	*m/z*	RT, s	Available MS^2 Spektrum, CE		
					10	20	40
pos	M192T258	4-MEAP-M (*N*-deethyl-)	192.1383	258	x	x	x
pos	M202T274	4-MEAP artifact (dehydro-)	202.1590	274	/	/	/
pos	M203T273	4-MEAP artifact (dehydro-) ^{13}C-isotope	203.1624	273	/	/	/
pos	M220T274	4-MEAP	220.1697	274	x	x	x
pos	M221T274	4-MEAP ^{13}C-isotope	221.1730	274	/	/	/
pos	M222T274	4-MEAP $^{13}C_2$-isotope	222.1762	274	/	/	/
pos	M222T277	4-MEAP-M (dihydro-)	222.1854	277	x	x	x
pos	M223T277	4-MEAP-M (dihydro-) ^{13}C-isotope	223.1886	277	/	/	/
pos	M236T193	4-MEAP-M (HO-)	236.1645	193	x	x	x
pos	M237T193	4-MEAP-M (HO-) ^{13}C-isotope	237.1679	193	/	/	/
pos	M238T193	4-MEAP-M (HO-) $^{13}C_2$-isotope	238.1711	193	/	/	/
pos	M238T195	4-MEAP-M (dihydro-HO-)	238.1801	195	x	x	x
pos	M250T197	4-MEAP-M (HOOC-)	250.1438	197	x	x	x
neg	M291T460	Unknown	291.1599	460	n.d.	n.d.	n.d.
pos	M298T318	Unknown	298.0801	318	n.d.	n.d.	n.d.
pos	M300T318	Unknown	300.0780	318	x	x	x
pos	M314T243	Unknown	314.0749	243	x	x	x
pos	M316T243	Unknown	316.0730	243	x	x	x

Table S5. Significant features of 4-MEAP detected using normal phase chromatography. Features ordered by mass per charge (*m/z*) and retention time. RT = retention time, CE = collision energy, pos = positive, neg = negative, x = available, / = not analyzed, n.d. = not detectable.

Ionization Mode	Feature	Identity	*m/z*	RT, s	Available MS^2 Spectrum, CE		
					10	20	40
pos	M105T288	4-MEAP artifact $[M+H-C_6H_{13}ON]^+$	105.0704	288	/	/	/
pos	M119T288	4-MEAP artifact $[M+H-C_6H_{15}N]^+$	119.0488	288	/	/	/
pos	M157T252	Unknown	156.8453	252	n.d.	n.d.	n.d.
pos	M159T252	Unknown	158.8433	252	n.d.	n.d.	n.d.
pos	M174T332	Unknown	174.1279	332	x	x	x
pos	M175T288	4-MEAP artifact $[M+H-C_2H_7N]^+$	175.1119	288	/	/	/
pos	M175T332	4-MEAP artifact $[M+H-C_2H_7N]^+$	175.1119	332	/	/	/
pos	M176T357	Unknown	176.1435	357	n.d.	n.d.	n.d.
pos	M188T335	4-MEAP-M (HO-) artifact $[M+H-CH_4O_2]^+$	188.1435	335	/	/	/
pos	M192T333	4-MEAP-M (*N*-deethyl)	192.1384	333	x	x	x
pos	M193T333	M193T333 ^{13}C-isotope	193.1418	333	/	/	/
pos	M194T358	Unknown	194.1541	358	x	x	x
pos	M195T358	M194T358 ^{13}C-isotope	195.1574	358	/	/	/
pos	M200T334	4-MEAP-M (HO-) artifact $[M+H-H_4O_2]^+$	200.1435	334	/	/	/
pos	M202T288	4-MEAP artifact (dehydro-)	202.1592	288	n.d.	n.d.	n.d.
pos	M203T288	4-MEAP artifact (dehydro-) ^{13}C-isotope	203.1626	288	/	/	/
pos	M204T313	4-MEAP artifact $[M+H-H_2O]^+$	204.1748	313	/	/	/
pos	M218T335	4-MEAP-M (HO-) artifact $[M+H-H_2O]^+$	218.1541	335	/	/	/
pos	M220T288	4-MEAP	220.1698	288	x	x	x
pos	M221T288	4-MEAP ^{13}C-isotope	221.1732	288	/	/	/
pos	M222T288	4-MEAP $^{13}C_2$-isotope	222.1763	288	/	/	/
pos	M222T314	4-MEAP-M (dihydro-)	222.1854	314	n.d.	n.d.	n.d.
neg	M223T213	Unknown	222.7644	213	n.d.	n.d.	n.d.
pos	M223T314	4-MEAP-M (dihydro-) ^{13}C-isotope	223.1887	314	/	/	/
pos	M234T283	Unknown	234.1854	283			
pos	M234T91	Unknown	234.1854	91	n.d.	n.d.	n.d.

Table S5. continued.

Ionization mode	Feature	Identity	*m/z*	RT, s	Available MS^2 Spektrum, CE		
					10	20	40
pos	M234T92	4-MEAP-M (oxo-)	234.1491	92	x	x	x
pos	M235T91	4-MEAP-M (oxo-) ^{13}C-isotope	235.1523	91	/	/	/
pos	M236T335	4-MEAP-M (HO-)	236.1647	335	x	x	x
pos	M237T75	Unknown	237.1680	75	n.d.	n.d.	n.d.
pos	M237T335	4-MEAP-M (HO-) ^{13}C-isotope	237.1680	335	/	/	/
pos	M238T335	4-MEAP-M (HO-) $^{13}C_2$-isotope	238.1713	335	/	/	/
pos	M238T367	4-MEAP-M (dihydro-HO-)	238.1802	367	x	x	x
pos	M239T367	4-MEAP-M (dihydro-HO-) ^{13}C-isotope	239.1836	367	/	/	/
pos	M250T113	Unknown	250.1803	113	n.d.	n.d.	n.d.
pos	M250T445	4-MEAP-M (HOOC-)	250.1439	445	x	x	x
pos	M251T117	Unknown	251.1472	117	n.d.	n.d.	n.d.
pos	M257T225	Unknown	257.2013	225	n.d.	n.d.	n.d.
pos	M272T91	Unknown	272.1048	91	n.d.	n.d.	n.d.
pos	M275T79	Unknown	275.1756	79	n.d.	n.d.	n.d.
neg	M276T213	Unknown	275.7595	213	n.d.	n.d.	n.d.
neg	M278T213	Unknown	277.7576	213	n.d.	n.d.	n.d.
pos	M298T275	Unknown	298.0802	275	n.d.	n.d.	n.d.
pos	M299T275	M298T275 ^{13}C-isotope	299.0835	275	/	/	/
pos	M301T275	M298T275 $^{13}C_2$-isotope	301.0815	275	/	/	/
pos	M314T312	Unknown	314.0751	312	x	x	x
pos	M315T229	Unknown	315.2435	229	n.d.	n.d.	n.d.
pos	M316T312	Unknown	316.0732	312	x	x	x
pos	M409T252	Unknown	409.1374	252	n.d.	n.d.	n.d.
pos	M442T252	Unknown	441.9522	252	n.d.	n.d.	n.d.
pos	M475T288	4-MEAP adduct $[2M+2H+Cl]^+$	475.3087	288	/	/	/
pos	M477T333	Unknown	477.2453	333	n.d.	n.d.	n.d.
pos	M479T358	Unknown	479.2609	358	x	x	x
pos	M521T335	Unknown	521.2715	335	n.d.	n.d.	n.d.

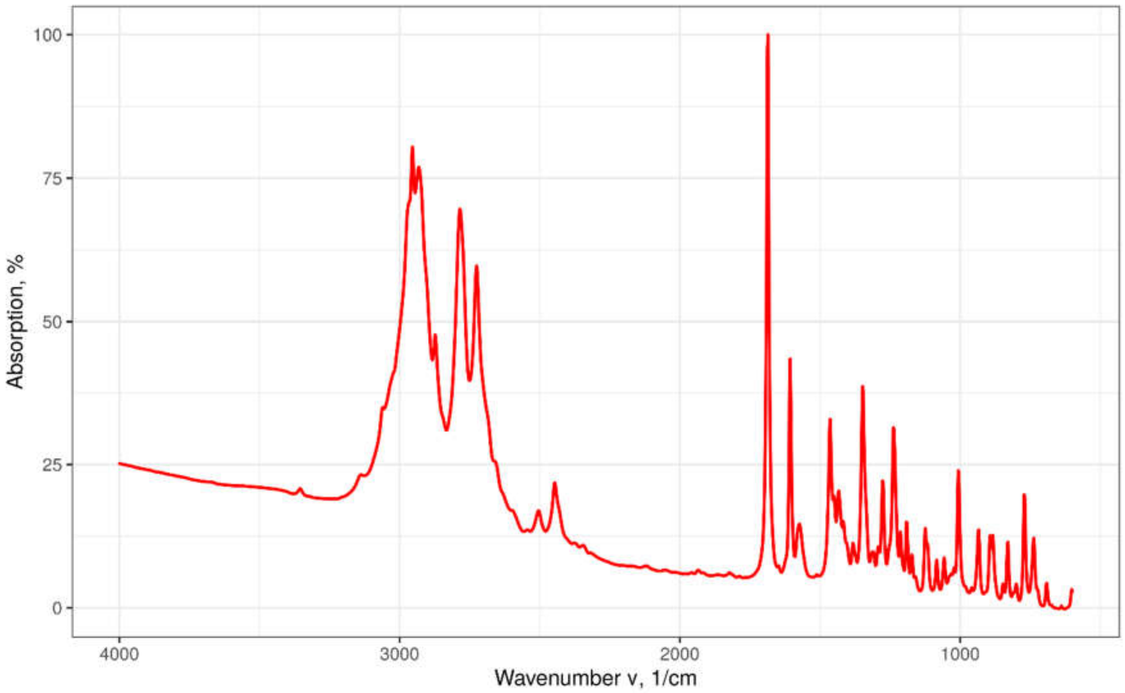

Figure S1. FT-IR spectrum of 4-MPD HCl (KBr pellet). The x-axis is displayed as wavenumbers (ν) in cm^{-1}, the y-axis as percentage of absorption. Peaks at ν: 3355, 3139, 3060, 2954, 2931, 2873, 2785, 2728, 2505, 2445, 2120, 1822, 1686, 1606, 1511, 1573, 1464, 1450, 1432, 1417, 1405, 1382, 1349, 1347, 1309, 1292, 1276, 1253, 1238, 1213, 1191, 1172, 1159, 1124, 1116, 1084, 1057, 1022, 1006, 981, 958, 933, 893, 883, 846, 829, 810, 771, 737, 723, 690, 638, 602.

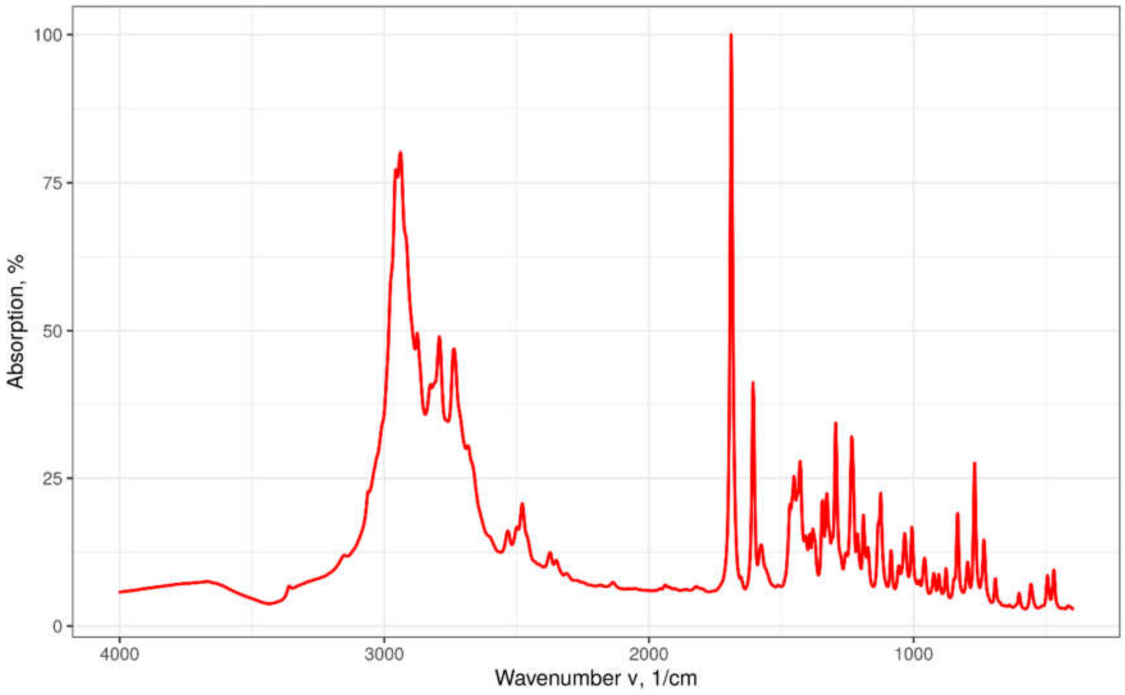

Figure S2. FT-IR spectrum of 4-MEAP HCl (KBr pellet). The x-axis is displayed as wavenumbers (ν) in cm^{-1}, the y-axis as percentage of absorption. Peaks at ν: 3360, 3151, 3060, 2956, 2938, 2875, 2826, 2792, 2737, 2533, 2499, 2478, 2136, 1822, 1689, 1606, 1575, 1512, 1467, 1452, 1428, 1407, 1392, 1380, 1344, 1328, 1307, 1294, 1255, 1234, 1211, 1189, 1172, 1134, 1124, 1085, 1057, 1033, 1006, 979, 958, 923, 904, 877, 846, 833, 796, 769, 735, 690, 638, 602.

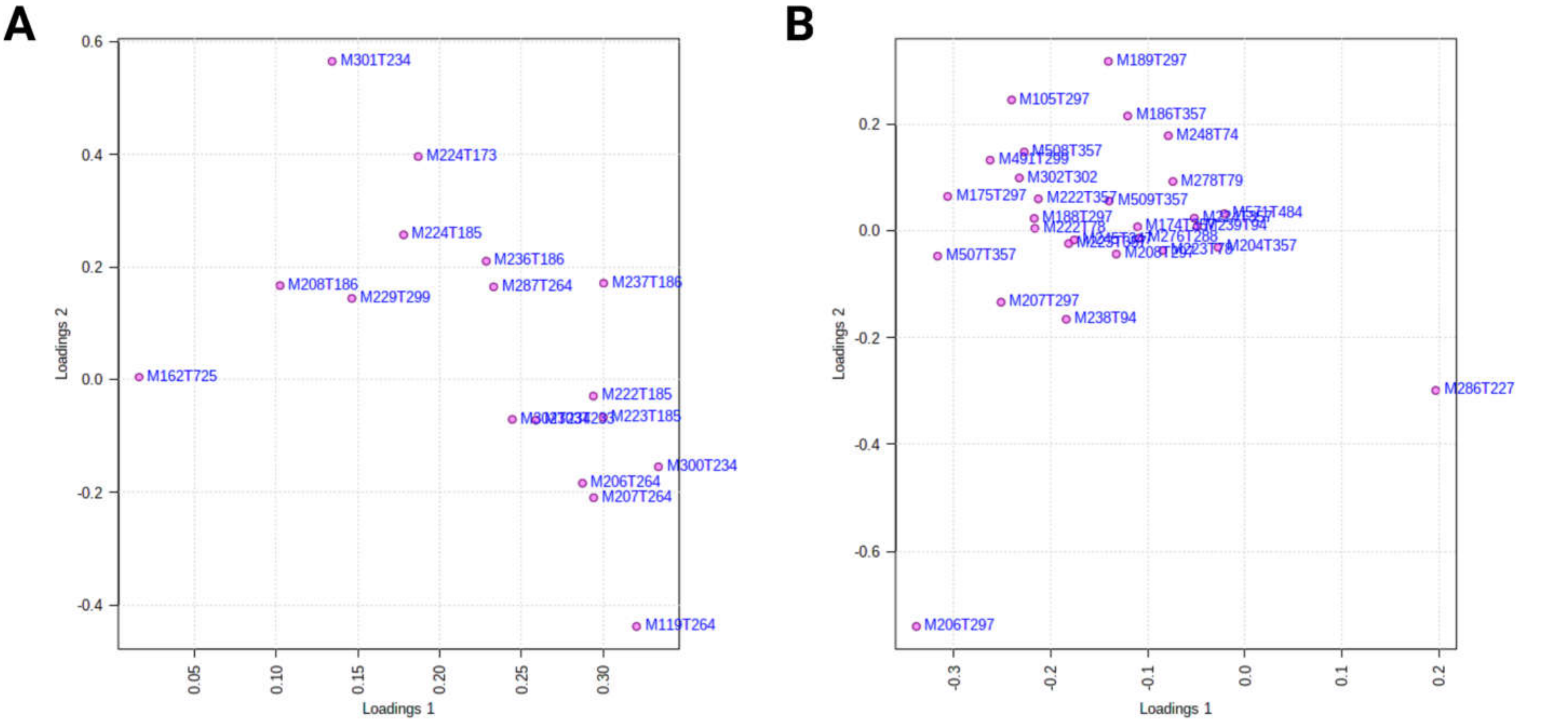

Figure S3. Loadings of principal component analysis. A = 4-MPD, reversed phase chromatography, positive ionization mode; B = 4-MPD, normal phase chromatography, positive ionization mode; C = 4-MPD, normal phase chromatography, negative ionization mode. D = 4-MEAP, reversed phase chromatography, positive ionization mode; E = 4-MEAP, normal phase chromatography, positive ionization mode; F = 4-MEAP, normal phase chromatography, negative ionization mode.

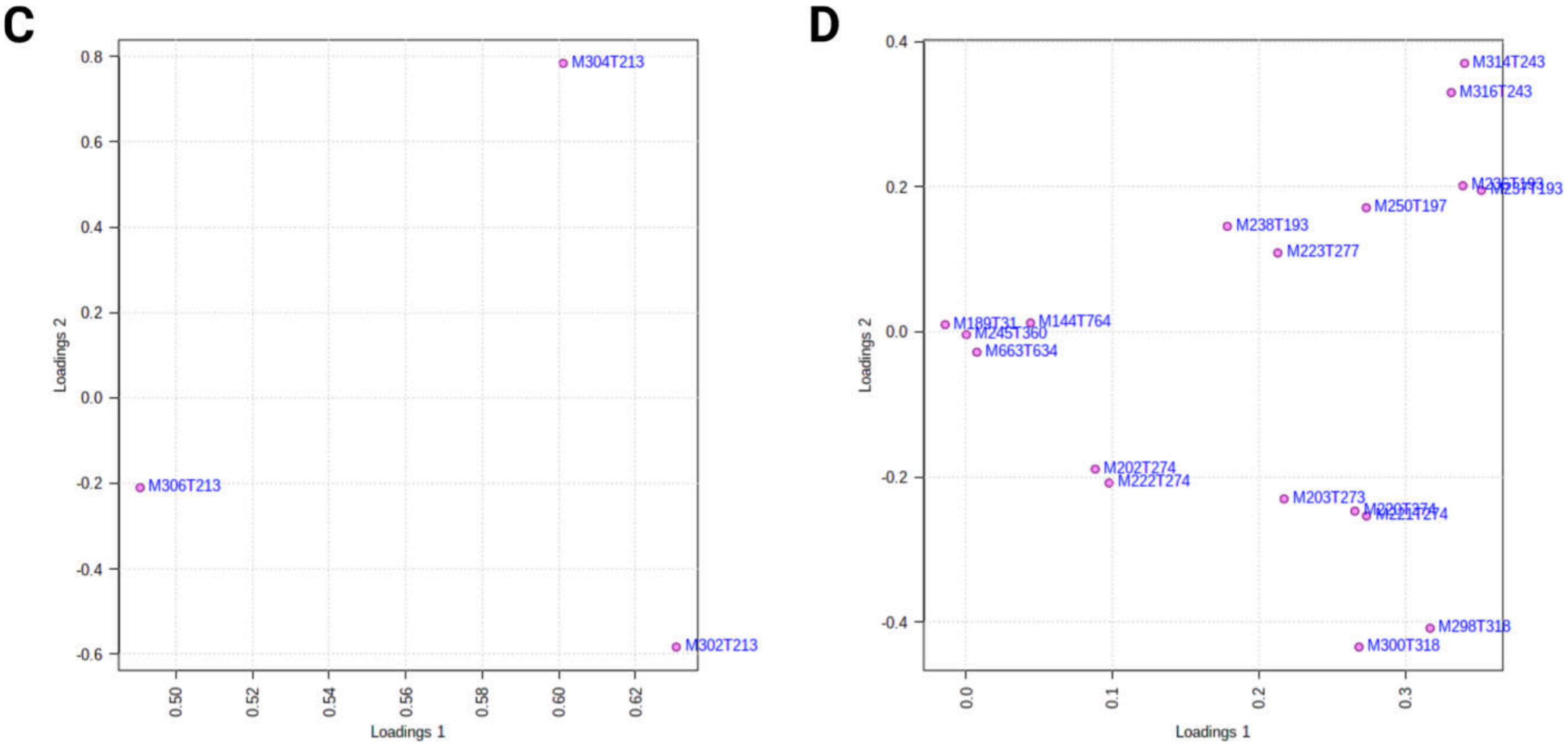

Figure S3. continued

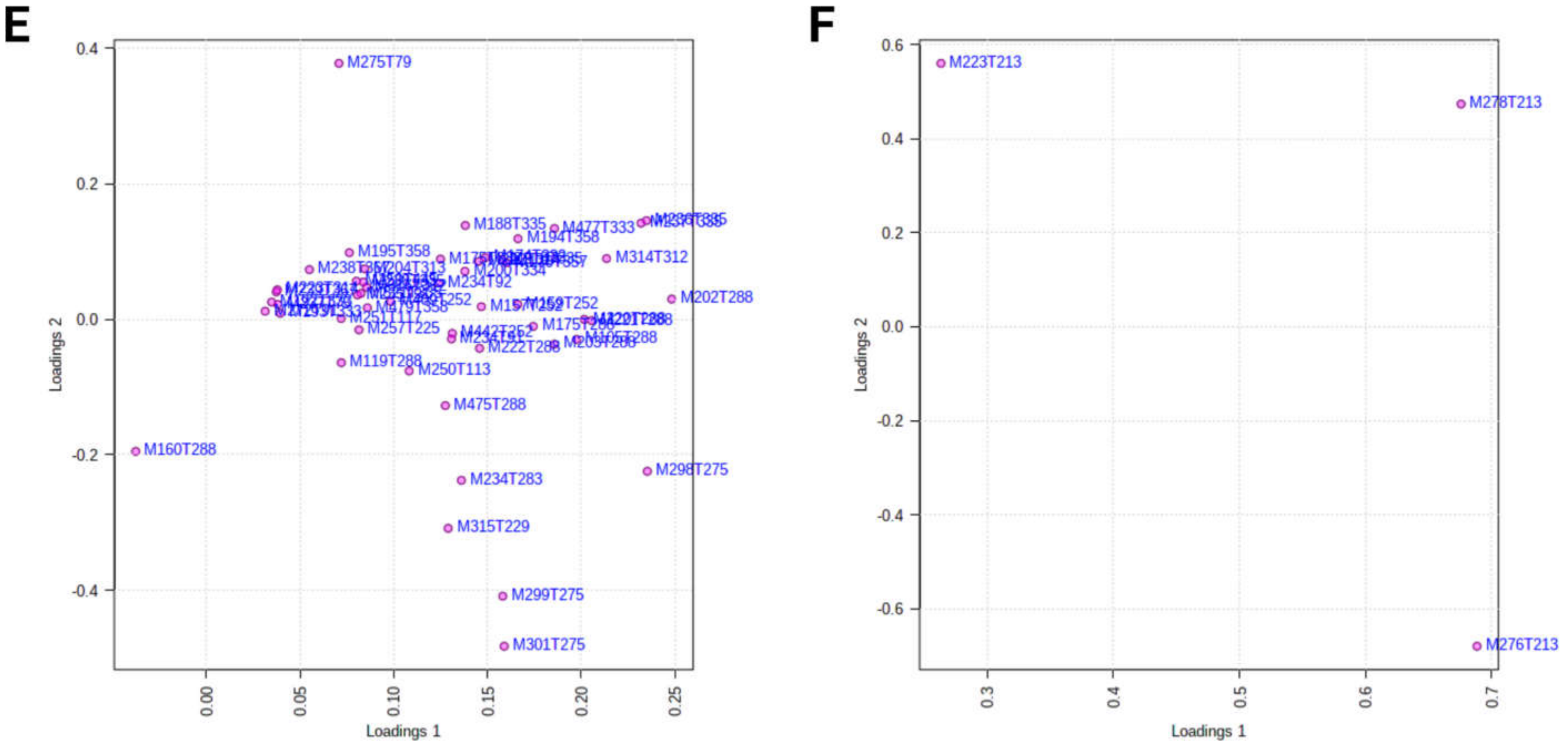

Figure S3. continued

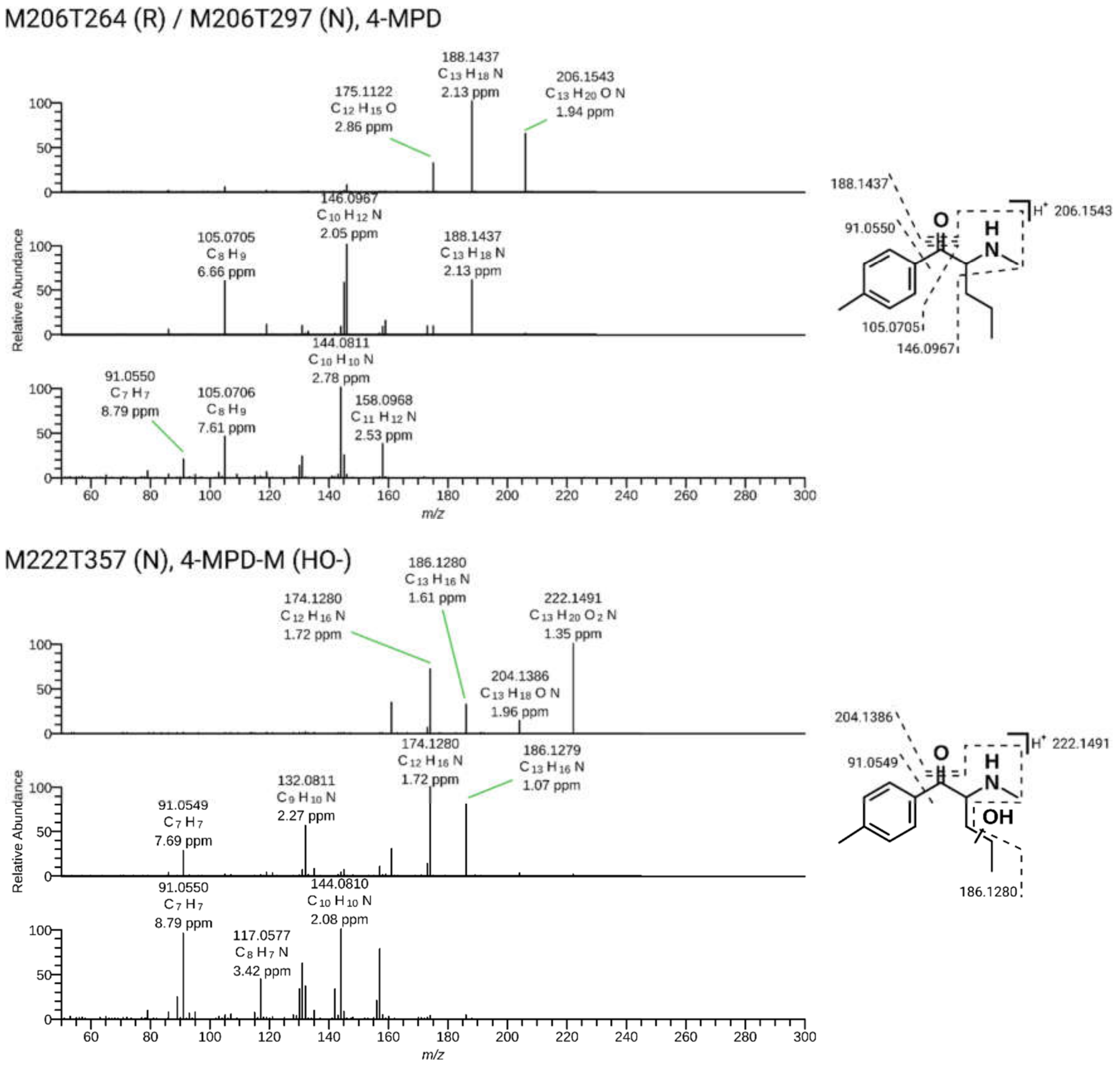

Figure S4. LC-HRMS/MS spectra of 4-MPD and its metabolites sorted by mass of their protonated molecule and retention time. Mass spectra after CE 10, 20, and 40 from top to bottom. Fragments with accurate mass, calculated elemental formula, and mass error value in parts per million (ppm). If a fragment occurred repeatedly after using different collision energies, the mass spectrometry scissors were labelled with the mass from the lowest collision energy. R = reversed phase chromatography, N = normal phase chromatography.

M222T78 (N), 4-MPD-M (hydroxylamine-)

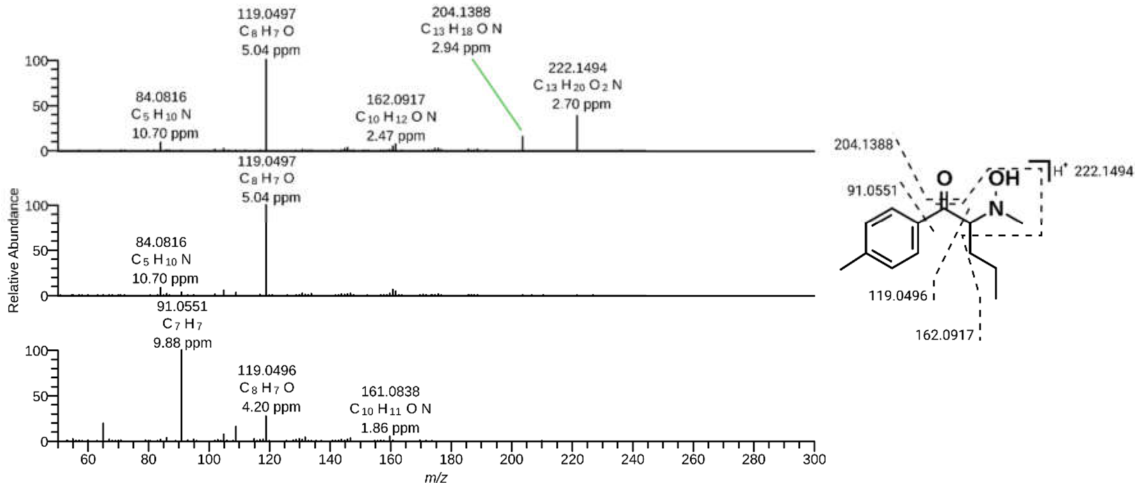

M224T173 (R), 4-MPD-M (dihydro-HO-)

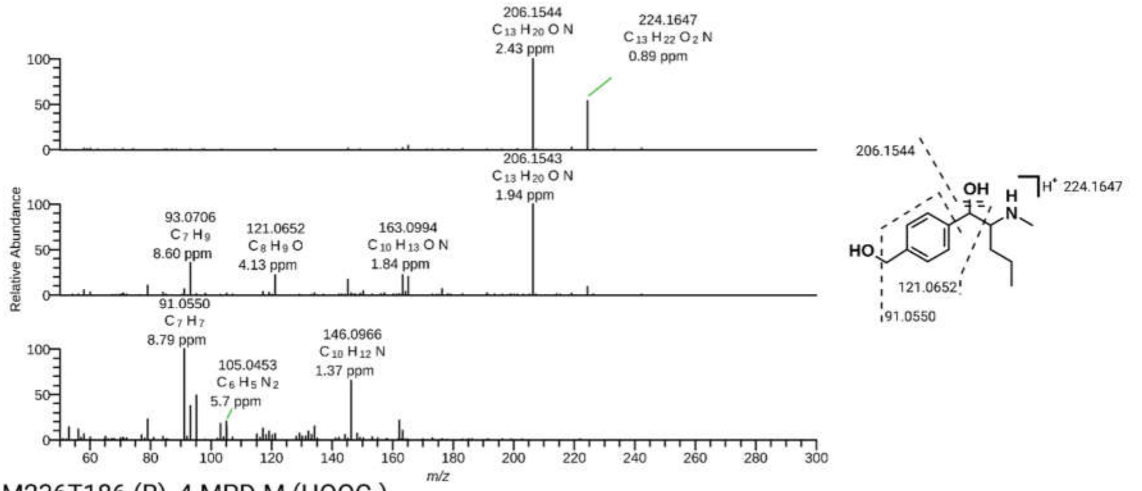

M236T186 (R), 4-MPD-M (HOOC-)

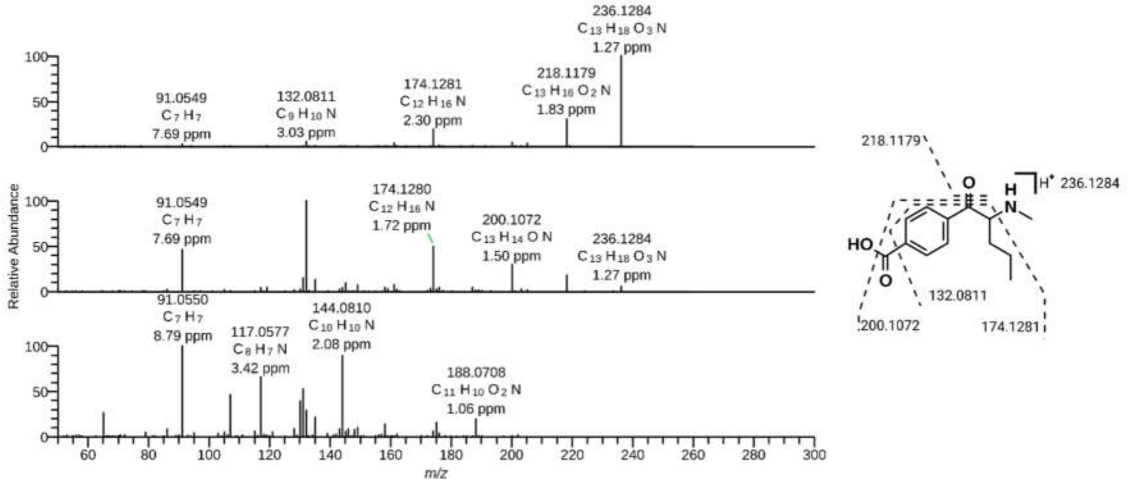

Figure S4. continued.

M238T94 (N), 4-MPD-M (di-HO-)

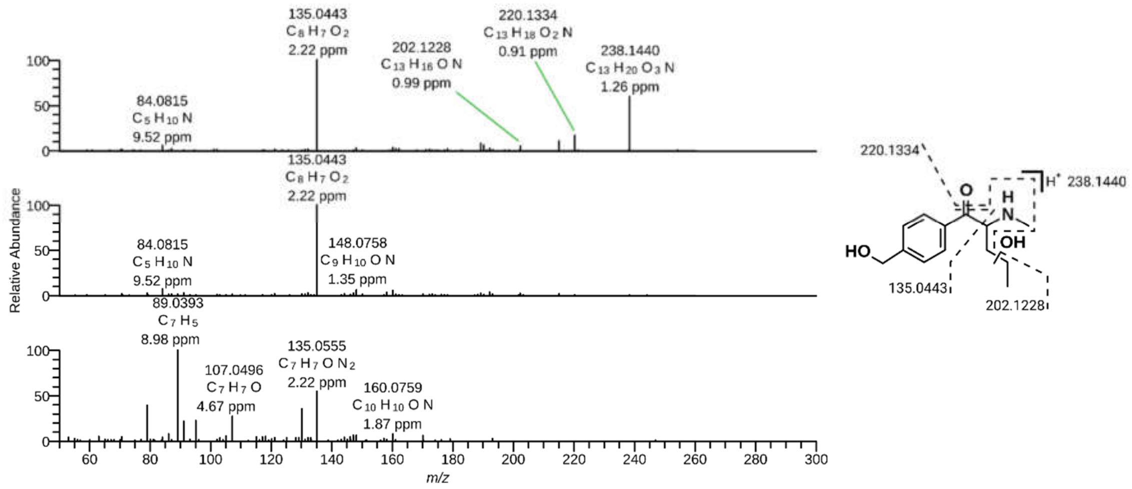

Figure S4. continued.

M192T258 (R) / M192T333 (N), 4-MEAP-M (*N*-deethyl-)

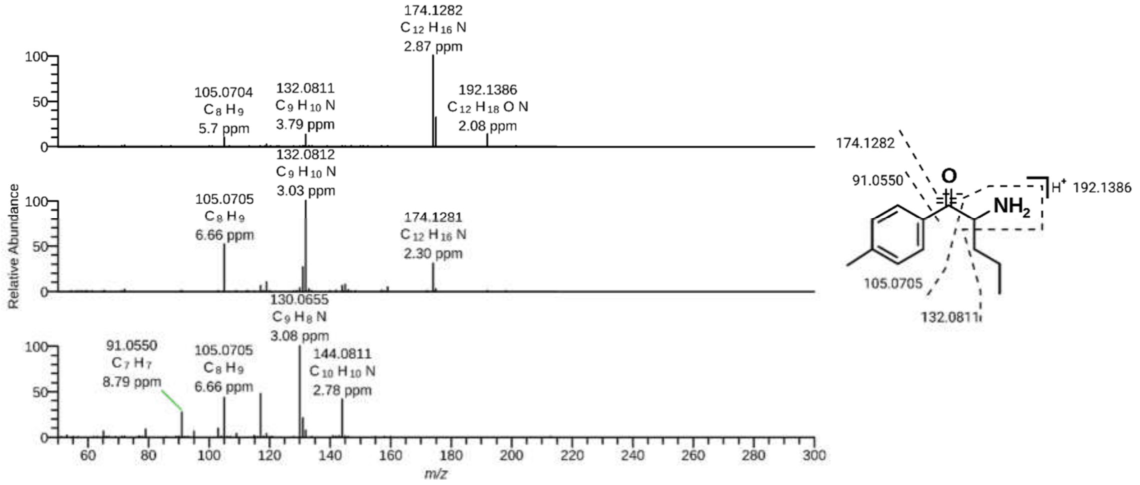

M220T274 (R) / M220T288 (N), 4-MEAP

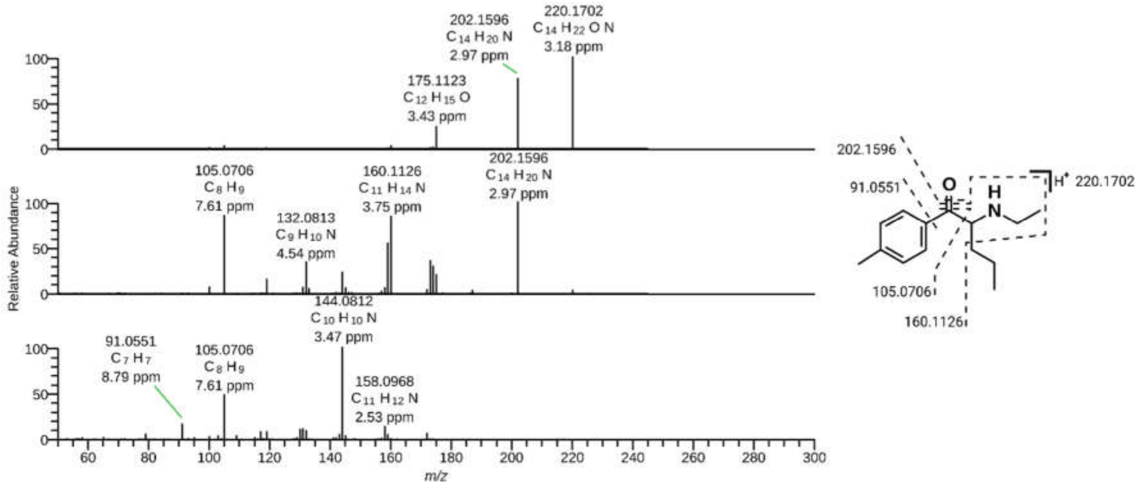

Figure S5. LC-HRMS/MS spectra of 4-MEAP and its metabolites sorted by mass of their protonated molecule and retention time. Mass spectra after CE 10, 20, and 40 from top to bottom. Fragments with accurate mass, calculated elemental formula, and mass error value in parts per million (ppm). If a fragment occurred repeatedly after using different collision energies, the mass spectrometry scissors were labelled with the mass from the lowest collision energy. R = reversed phase chromatography, N = normal phase chromatography.

M222T277 (R) / M222T314 (N), 4-MEAP-M (dihydro-)

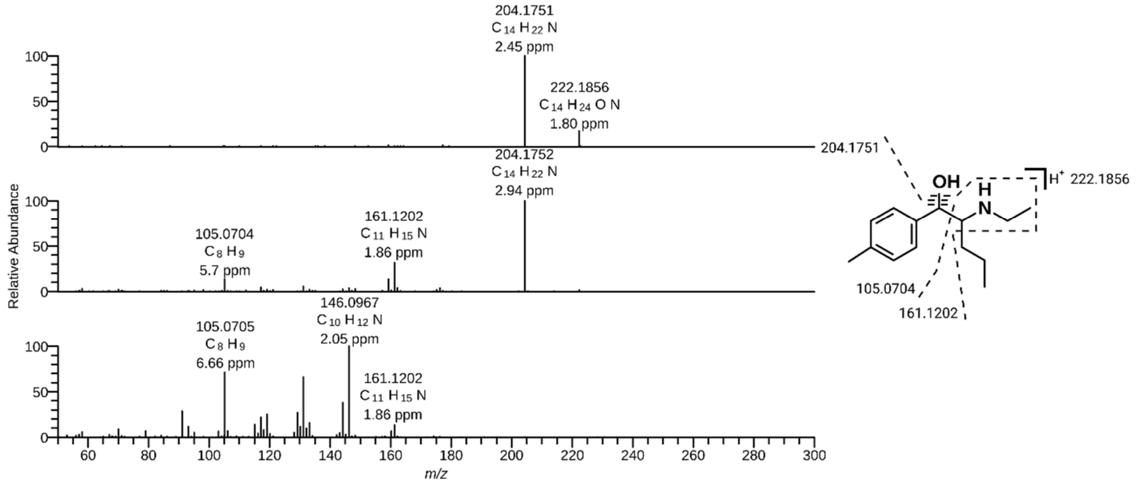

M234T92 (N), 4-MEAP-M (oxo-)

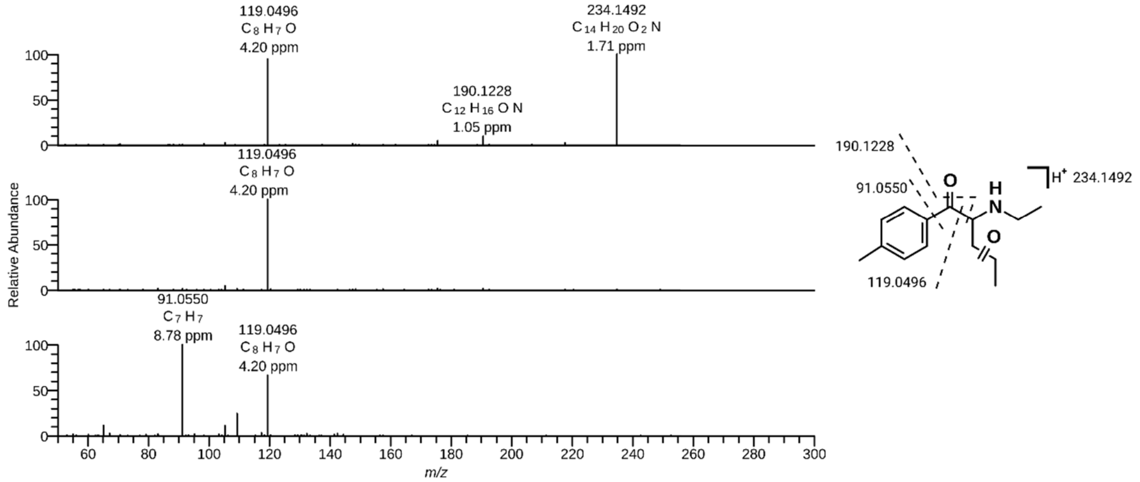

M236T193 (R) / M236T335 (N), 4-MEAP-M (HO-)

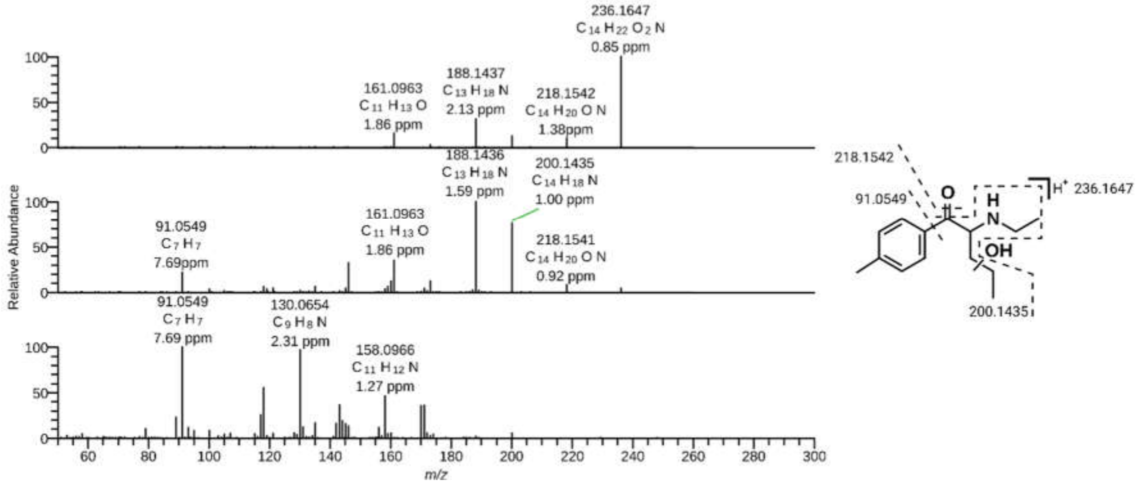

Figure S5. continued.

M238T195 (R) / M238T367 (N), 4-MEAP-M (dihydro-HO-)

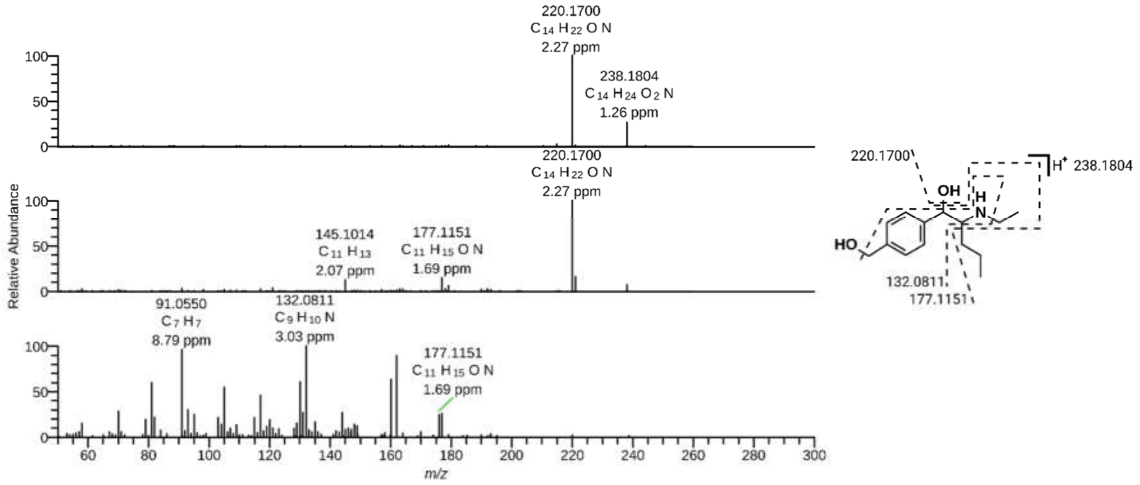

M250T197 (R) / M250T445 (N), 4-MEAP-M (HOOC-)

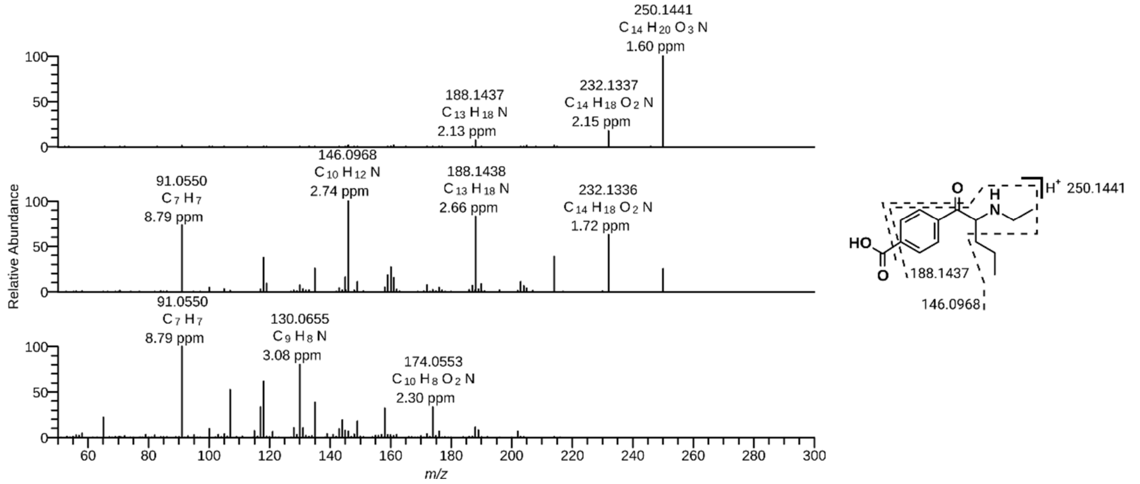

Figure S5. continued.

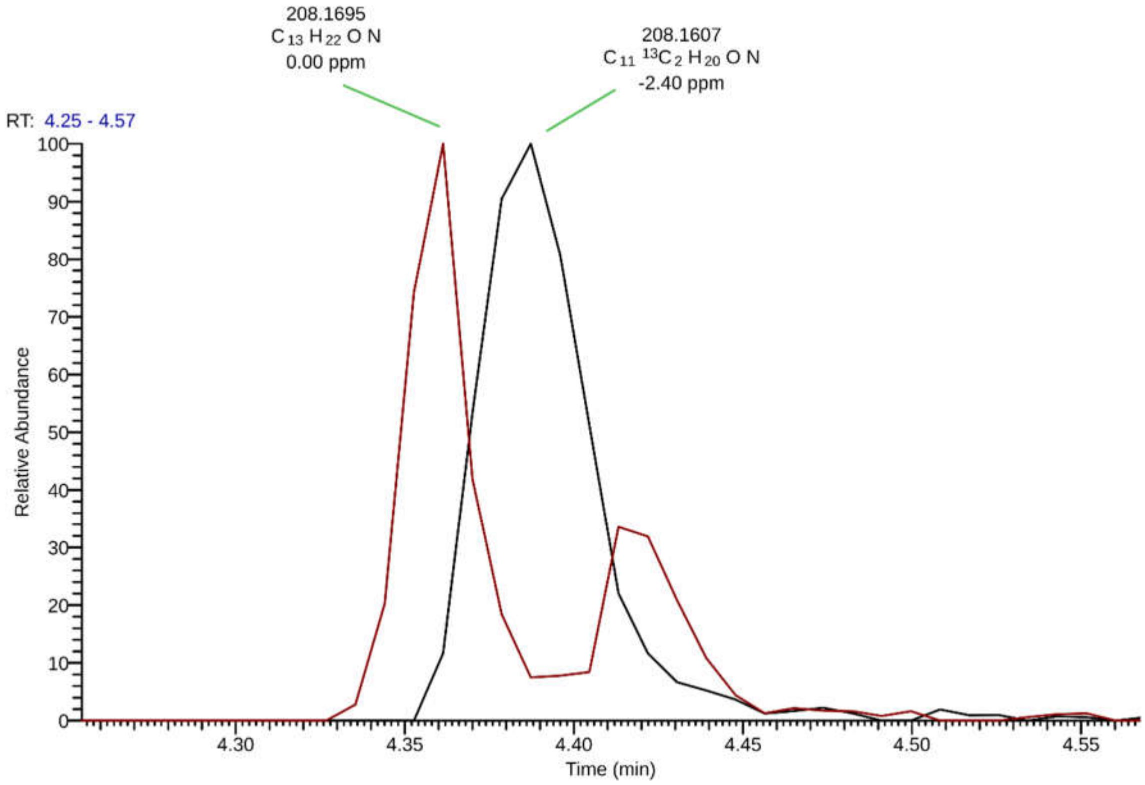

Figure S6. Extracted ion chromatogram of two peaks assumed to be 4-MPD isotope ($^{13}C_2$) and 4-MPD-M (dihydro-) taken from sample High 1 after analysis using reversed phase chromatography and positive ionization.
